# Upon a Disease of the Stomach, Which Produces a Well-Defined Perforation in Its Tunics, without Softening of Their Surrounding Structure

**Published:** 1828-11

**Authors:** C. H. Ebermaier


### DISEASES OF THE STOMACH.

*Upon a Disease of the Stomach, which produces a well-defined Perforation in its Tunics, without Softening of their surrounding Structure.* By Dr. C. H. Ebermaier.

(Concluded from page 308.)

Case VII. (related by M. Trinnius.)—A man, apparently in good health, was suddenly attacked, after exposure to cold and rain, with a violent pain in the region of the stomach. His suffering gradually diminished, but he remained subject to frequent attacks of spasm in the stomach. His complaints, however, continued so trifling for the space of a year, that he did not, until the expiration of that time, seek for medical assistance. At this period he was robust in appearance, and, with the exception of slight spasmodic affections of the stomach, which did not, however, cause any local tenderness or increase of sensibility, he enjoyed good health. The digestive functions were undisturbed. He was quickly relieved by the use of the oxyde of bismuth. In the course of a few months, the cardialgic affection returned with increased severity. Any irregularity in diet obviously added to the sufferings of the patient. Antispasmodics produced no good effect. He had occasional intervals of ease, during which the stomach was not disturbed by food, and he was able to take moderate exercise.

About a fortnight after his relapse, he rode out one morning in a carriage, and ate heartily of salt meat. On returning home, he was seized with the most violent pains, with great agitation, and difficulty of breathing. The surface of the belly appeared to be drawn towards the spine; the face and extremities were pale and cold. Great pain in the abdomen; tenesmus. For the first time he vomited a quantity of tenacious mucus. He complained of a sense of fulness in the lower part of the belly: it was not, however, tense or hard. The pulsations at the wrist were too rapid to be numbered. The faculties were undisturbed. The slightest motion gave rise to the sensation of some heavy body rolling about in the abdomen. In a few hours he died.

*Dissection.*—A considerable quantity of chocolate-coloured fluid escaped from the abdomen. The stomach was pale and shrunk, and near the pylorus there was found an aperture of a circular form, about an inch in diameter. A more accurate examination detected several points of preternatural adhesion with the surrounding parts. In other respects, the stomach and the other viscera were perfectly healthy.

Case VIII.—A man, twenty years old, who had been subject to dyspeptic attacks after eating, was attacked suddenly with very violent spasmodic pain in the stomach. He threw himself upon a sofa, his body bent with suffering, and uttering dreadful cries upon the least motion. The pulse was not to be felt. At the end of a few hours he died, in perfect possession of his faculties.

*Dissection.*—A good deal of air escaped from the abdomen. In the cavity was found a mixture of solid and liquid food, that had escaped from the stomach. The stomach was shrunk, and upon its anterior surface, near the lesser curvature, about two inches from the pylorus, was found an opening with smooth edges, which appeared as if it had been made with a punch. It was rather oval than round, nine lines in length, and six in diameter. Nearly opposite this aperture, on the posterior part of the stomach, there was a small round spot of mortification, which appeared on the point of giving way. There was no appearances of recent inflammation, or determination of blood. All the other viscera were healthy.

Case IX.—This case was communicated to Dr. E. by M. Thevissen. It is particularly interesting, inasmuch as it proves that a similar kind of disorganization may take place in other organs, the structure of which admits of the formation of perforations, with fatal extravasation of their contents.—An unmarried woman, thirty-three years of age, whose health had never been disturbed by any serious illness, suddenly complained of very severe pain in the lower part of the belly. No cause could be assigned for the attack. She imagined it was possible that her attack might be dependent upon suppression of the menses, which had existed for four months. The lower belly was highly sensible to the slightest pressure. Thirst excessive. Extremities covered with sweat, and cold as marble. Countenance anxious, and with a yellowish tinge. Pulse small and quick. Abdominal inflammation was suspected, and a severe antiphlogistic treatment was instituted without any benefit. The pains increased in severity. Frequent vomitings of a dark brown substance. The patient gradually sunk, and died the night after she was attacked. She had had no motion, nor passed any urine, during her short but severe illness.

*Dissection.*—A considerable quantity of fluid escaped from the abdomen, of an urinous smell. There was no trace of gangrene nor of inflammation. All the viscera were healthy. The uterus contained a four-months' fœtus. On the posterior side of the bladder, about the middle of its longitudinal diameter, a circular perforation was found, about two lines in diameter. The edges of this opening were neither gangrenous, inflamed, nor hard; they were as smooth as if a portion had been removed by a punch. In every other part the bladder was perfectly healthy.

After having cited these very interesting examples, Dr. E. takes a brief view of the opinions which other physicians have entertained upon the subject, and concludes by stating his own sentiments.

Gerard conceives that the perforations of the stomach cannot be attributed, in general, to any acrid matter. If such were the fact, the organ would be corroded to a greater extent. These apertures are usually found in patients apparently in good health, or who at least are not suffering under any malady alarming in itself. He imagines that death takes place in consequence of rupture of the stomach from gangrene, ulceration, or abscess; and he suggests that a small abscess may be slowly developed in the tunics of the stomach, which may entirely destroy the coats. A case related by Lieutaud renders this probable. He found pus between the membranes of the stomach, in the body of a woman who had long suffered from cardialgia.

Chaussier derives all the perforations from scirrhous affections, or from suppuration. He rejects the spontaneous digestion of the stomach admitted by Hunter, and also the action of worms. He supposes that the apertures arise from a morbid process of ulceration, which may be either acute or chronic. Although there is, in the first instance, no chemical alteration in the humors, still the cause depends upon a particular irritation of the solids, in consequence of which the humors acquire a dissolvent property, as is proved by the lint placed upon ulcers being frequently perforated or dissolved. It is impossible to characterise this morbid process by its external signs or by its essence, because it takes place in the tissue of the organs, in the extremities of the lymphatic, vascular, and nervous systems. It is, in fact, known only by its results. It is the opposite of the process of nutrition, which is as completely hidden from our observation. When it attacks a part, its blood-vessels are gradually multiplied, and appear injected. An ichorous fluid is discharged, which attacks the tissue, and destroys every part it touches. The spots and perforations of the stomach are different degrees of one and the same malady. Sometimes the destructive process is suddenly established, in the space of a few hours, and in healthy subjects. It more frequently occurs after some days' illness.

Henke is of opinion that these spontaneous perforations of the stomach are identical with the disease known in Germany by the name of gelatiform softening of that viscus, and which is observed principally in children, and that it is preceded by inflammation of the stomach, of a more or less acute nature.

Desgranges maintains that, in the case he saw, the violent contractions of the stomach, always acting upon a single point, produced a mechanical lesion of the part.

According to Rauch, perforations of the stomach occur in four different forms: 1. Ulcers with their edges, which are callous, or inflamed or sphacelated, and that then the tunics of the viscus are gradually destroyed. 2. Destruction of a perfectly healthy tissue, with irregular and fringed edges, more or less inflamed: such perforations arising from the action of gas, efforts at vomiting, &c. 3. Round holes with smooth edges, without suppuration, gangrene, inflammation, softening, or thickening. Also a gradual thinning, with local absorption of the coats. 4. Gelatiniform softening.

Various other opinions are glanced at, but they do not refer to those round perforations of the stomach, the edges of which are smooth and not thinned.

Dr. E. is of opinion that the cases he has related prove the existence of some common morbific cause, which could produce so striking an uniformity in the appearances seen upon dissection. He observes—1. That in every case the disease was extremely slow, being gradually developed in the course of several years. 2. In no instance was the nature of the malady suspected by the physicians. The symptoms were so obscure in some instances, that an affection of the stomach was never thought of. The derangements of the digestive functions were considered to be sympathetic. The fatal termination of the disease was never anticipated. Death sometimes occurred unexpectedly, almost in the midst of apparent health. 3. The disease continued uninterruptedly, without any perfect intervals, as is frequently the case in true nervous cardialgia, although occasionally in so slight a degree that the patient considered himself in health. Severe pain did not usually occur until the last days of the patient's existence, and not always even at that time. The previous pains were slight, limited to a dull sensation of pressure in the precordial region, and to slight spasms. 4. Cachexy never followed this long train of symptoms. Although vomiting frequently occurred, the strength of the patient did not appear diminished, nor did his external appearance indicate the existence of disease. Emaciation occurred only in the case related by Rauch, and that was an example of a complicated malady. In every other no hectic fever was observed, and death was neither the result of exhaustion of the vital powers nor want of nutrition: it was sudden, and caused by the extravasation of the contents of the stomach, without which the patient might have continued to live. 5. The perforations were always found in the pyloric region, or near it. 6. The most attentive examination could not in any case detect the least vestige of inflammation or suppuration of the other parts of the stomach. The tunics of that organ were perfectly healthy, except in the spot perforated, and rather pale than red. 7. The appearance of the perforation was always the same. Approaching a perfectly round form, and almost always of considerable extent, it penetrated uniformly all the coats of the stomach, so that the portion removed appeared to have been taken away in a very regular manner. The surrounding parts were never softened, nor the edges thinned. There was generally perceived around the opening a tumefied induration, but not tuberculous nor cartilaginous. It was regular, and lost insensibly in the healthy parts.

Dr. E. considers the cases he has related particularly interesting, and calculated to throw some light upon the true nature of these perforations, on account of the adventitious and thickened tissue which surrounded the apertures. It follows that the rupture could not have arisen from the thinness or local weakness of the part, but that it depended upon a regular and uniform process, continuing without interruption from the commencement of the disease.

It may then be concluded, that these regular perforations of the stomach are never the accidental or mechanical result of spasm. That the disease does not consist in scirrhus or cancer of the stomach. That it is not the termination of an ordinary chronic inflammation. Lastly, that it does not result from "ramollissement" of the parietes of the stomach.

